# An Indirect Defence Trait Mediated through Egg-Induced Maize Volatiles from Neighbouring Plants

**DOI:** 10.1371/journal.pone.0158744

**Published:** 2016-07-08

**Authors:** Daniel M. Mutyambai, Toby J. A. Bruce, Johnnie van den Berg, Charles A. O. Midega, John A. Pickett, Zeyaur R. Khan

**Affiliations:** 1 Habitat Management Programme, International Centre of Insect Physiology and Ecology, P.O Box 30–40305, Mbita, Kenya; 2 School of Biological Sciences, North-West University, Potchefstroom, 2520, South Africa; 3 Biology Department, South Eastern Kenya University, P.O. Box 170–90200, Kitui, Kenya; 4 Department of Biological Chemistry, Rothamsted Research, Harpenden, Herts, AL5 2JQ, United Kingdom; Pennsylvania State University, UNITED STATES

## Abstract

Attack of plants by herbivorous arthropods may result in considerable changes to the plant’s chemical phenotype with respect to emission of herbivore-induced plant volatiles (HIPVs). These HIPVs have been shown to act as repellents to the attacking insects as well as attractants for the insects antagonistic to these herbivores. Plants can also respond to HIPV signals from other plants that warn them of impending attack. Recent investigations have shown that certain maize varieties are able to emit volatiles following stemborer egg deposition. These volatiles attract the herbivore’s parasitoids and directly deter further oviposition. However, it was not known whether these oviposition-induced maize (*Zea mays*, L.) volatiles can mediate chemical phenotypic changes in neighbouring unattacked maize plants. Therefore, this study sought to investigate the effect of oviposition-induced maize volatiles on intact neighbouring maize plants in ‘Nyamula’, a landrace known to respond to oviposition, and a standard commercial hybrid, HB515, that did not. Headspace volatile samples were collected from maize plants exposed to *Chilo partellus* (Swinhoe) (Lepidoptera: Crambidae) egg deposition and unoviposited neighbouring plants as well as from control plants kept away from the volatile emitting ones. Behavioural bioassays were carried out in a four-arm olfactometer using egg (*Trichogramma bournieri* Pintureau & Babault (Hymenoptera: Trichogrammatidae)) and larval (*Cotesia sesamiae* Cameron (Hymenoptera: Braconidae)) parasitoids. Coupled Gas Chromatography-Mass Spectrometry (GC-MS) was used for volatile analysis. For the ‘Nyamula’ landrace, GC-MS analysis revealed HIPV production not only in the oviposited plants but also in neighbouring plants not exposed to insect eggs. Higher amounts of EAG-active biogenic volatiles such as (*E*)-4,8-dimethyl-1,3,7-nonatriene were emitted from these plants compared to control plants. Subsequent behavioural assays with female *T*. *bournieri* and *C*. *sesamiae* parasitic wasps indicated that these parasitoids preferred volatiles from oviposited and neighbouring landrace plants compared to those from the control plants. This effect was absent in the standard commercial hybrid we tested. There was no HIPV induction and no difference in parasitoid attraction in neighbouring and control hybrid maize plants. These results show plant-plant signalling: ‘Nyamula’ maize plants emitting oviposition-induced volatiles attractive to the herbivore’s natural enemies can induce this indirect defence trait in conspecific neighbouring undamaged maize plants. Maize plants growing in a field may thus benefit from this indirect defence through airborne signalling which may enhance the fitness of the volatile-emitting plant by increasing predation pressure on herbivores.

## Introduction

In their natural habitats, plants live in complex communities comprising herbivores, pollinators, microbes, carnivores and neighbouring conspecific and other plants [1–3]. These plants are thus under selection pressure to maximize fitness within a complex setting of biotic interactions, with positive and negative outcomes [4]. As such, plants have evolved a diverse array of defence strategies against the attacking organisms, including herbivores and parasitic plants [5]. In particular, plants respond to herbivore attack through production of a number of chemical signals known as herbivore-induce plant volatiles (HIPVs), which have direct and/or indirect effects on the attacking herbivore. Directly, these chemical cues negatively affect the physiology or behaviour of the herbivore, either as toxins, digestibility reducers or deterrents [6, 7]. Indirectly, plants use these HIPVs to attract natural enemies of the herbivores, as well as increase the foraging success of these natural enemies, thereby facilitating improved control of herbivores [8,9].

HIPVs play a role in multitrophic community interactions by facilitating communication between the infested plant and natural enemies of the attacking herbivores, and also warning undamaged neighbouring plants of the same or another species, of the impending attack [10–12]. They also systemically facilitate communication between different parts of the same plant (intraplant signalling) [13–16]. The HIPVs are emitted not only from the infested plant parts but also systematically from uninfested parts of the plant which increases the detectability of the signal cues [4, 17–19]. However, different plant species produce entirely different blends of HIPVs and even within one plant species, there can be genotypic variation in HIPV production [20–22].

Undamaged plants that can activate and tailor their defences according to information derived from their attacked neighbouring plants may gain a selective advantage over plants that are unable to make use of the signal cues [23]. Evidence of plants being capable of ‘eavesdropping’ on airborne signals has been documented [24–28, 8, 29, 30, 23]. HIPVs can immediately induce defence in neighbouring plants at artificially high levels [31] while at the same time, physiologically relevant levels of induced volatile organic compounds (VOCs) can prime plants to prepare themselves for future pest and pathogen attack [31]. Perceived plant volatiles can also have physiological effects on the receiving plant as evidenced by changes in the transcription of defence-related genes [11, 32, 33]. Exposure of plants to herbivore-induced volatile organic compounds can result in changes in the abundance of phyto-hormones [34, 35] and increase production of defence-related metabolites such as terpenoids [35, 36], proteinase inhibitors [30] and phenolic compounds [30]. These plant defence strategies can be exploited in the management of injurious pests such as cereal stemborers.

Effective production of maize and other cereal crops is severely constrained by cereal stemborer pests, with the indigenous species, *Busseola fusca* Füller (Lepidoptera: Noctuidae) and the invasive *Chilo partellus* Swinhoe (Lepidoptera: Crambidae) being the most damaging in eastern Africa [37]. Effective management of these pests however remains elusive for smallholder farmers due to challenges posed by the boring activity of the larvae, the limited resources available to the farmers making chemical control methods unaffordable [38], and lack of empirical evidence of effectiveness of some of the cultural control methods [39]. Plant signalling through HIPVs or their variants thus represents an opportunity for effective control of stemborer pests. HIPVs are produced by plants long after damage has been inflicted to the plant by feeding larvae [40]. However, recent studies indicate that some plant genotypes such as *Pinus sylvestris*, *Zea mays*, teosinte (*Zea species*) are able to respond to the initial stage of herbivore attack (egg deposition) by emission of volatile organic compounds that are attractive to certain natural enemies [41, 42, 19, 43–45] or suppression of constitutive HIPV emission [46]. Investigations involving maize-herbivore-natural enemy tritrophic interactions have shown that egg deposition by cereal stemborers can induce volatile emission in certain maize varieties. These volatiles attract natural enemies and deter further herbivore colonization [19, 43–45]. This is seen as a preventative defence mechanism since parasitoids are recruited in advance, before the phytophagous larvae hatch and cause damage to plants. However, it is not known whether these oviposition-induced volatiles can mediate the same indirect defence in neighbouring unattacked maize plants. This current study thus sought to determine 1) effects of oviposition-induced volatiles on neighbouring unattacked maize plants; 2) subsequent effect on stemborer parasitoids of any volatiles from neighbouring unattacked maize plants. This would provide insights into the potential role of plant signalling in enhancing management of stemborer pests, particularly for the resource-constrained smallholder farmers in sub-Saharan Africa.

## Materials and Methods

### Study site

This study was carried out at Thomas Odhiambo Campus, Mbita Point (0° 25’S, 34° 12’E, 1200 m above sea level), a field station of the International Centre of Insect Physiology and Ecology (*icipe*) located on the shores of Lake Victoria in western Kenya where cereal stemborers are a serious constraint to maize cultivation.

### Plants

‘Nyamula’, a local maize landrace that has been shown to emit oviposition-induced volatiles upon stemborer egg deposition [43, 45] and hybrid maize variety, HB515, previously shown not to emit volatiles up on egg deposition [45] were used in this experiment. Seeds, obtained from local smallholder farmers in the Mbita region for ‘Nyamula’ and Western Seed Company, Kitale, Kenya for HB515, were planted individually in pots filled with fertilised soil in an insect-proof screen house under natural conditions (25°C, 65%RH; 12L: 12D). The seedlings were used in experiments when they were 3–4 weeks old, approximately 45 cm tall.

### Insects

*Chilo partellus* moths used in this study were obtained from the insect mass rearing unit at the *icipe*-Thomas Odhiambo campus. The larvae originated from field-collected stemborers, principally from sorghum *Sorghum bicolor* (L.) Moench fields in the Mbita region in western Kenya. Larvae were reared on a semi-synthetic diet containing sorghum leaf powder [47]. Field collected egg parasitoids, *Trichogramma bournieri* Pintureau & Babault (Hymenoptera: Trichogrammatidae) and larval parasitoids, *Cotesia sesamiae* Cameron (Hymenoptera: Braconidae) were reared on stemborer eggs and larvae respectively, using methodologies described by Overholt [48]. The insects were maintained at 24 ± 3°C, 70 ± 5% RH, 12L: 12D. The mass-reared culture was infused with a field-collected population every three months to avoid genetic decay and maintain the original behavioural characteristics of the species. Naive mated female moths and parasitoids obtained from second to third generation of the original field collected culture were used in the experiments.

### Volatile collection

Volatile compounds were collected using headspace sampling [49] from whole maize plants subjected to the following treatments: with stemborer eggs (inducing plants), without stemborer eggs but exposed to egg exposed plants (induced plants) and, without stemborer eggs and not exposed to plants with eggs (control plants). Prior to volatile collection, seedlings for oviposition were placed in oviposition cages (80 X 40 X 40 cm) into which five gravid naive female moths were introduced and kept overnight for oviposition. A wad of cotton wool (10 cm in diameter) moistened with water was placed into the cage for the moths to feed on the water from wet cotton wool. The following day, 20 egg exposed landrace maize plants were moved into an insect-proof screen house and arranged in two rows, 60 cm apart and 30cm between the plants to act as the inducing plants. Ten unexposed maize plants, five each for ‘Nyamula’ and HB515, and of the same age were then introduced between the two rows and placed 30cm apart. The set up was left for three days. Control plants were kept inside similar cages but without *C*. *partellus* moths in a similar insect-proof screen house. Volatiles were collected from these plants over a period of 48 hours, starting at the last two hours of photophase. Maize leaves were enclosed in polyethyleneterephthalate (PET) bags (3.2 L, ∼ 12.5 mm thick) heated to 150°C before use and were fitted with a swagelock inlet and outlet ports. Charcoal-filtered air was pumped (600 mL min^-1^) through the inlet port and volatiles collected on Porapak Q (0.05g, 60/80 mesh; Supelco Inc. Bellefonte, PA, USA) filters inserted into the outlet through which air was drawn at 400 mL min^-1^. Elution of the entrained volatiles was done using 0.5 mL dichloromethane and eluted samples stored in tightly capped microvials in a -20°C freezer prior to bioassays and further analysis. Entrainments from both plants with eggs (inducing), induced and control plants were replicated five times, and each plant was used only once.

### Behavioural bioassay

Responses of the parasitoids to plant derived volatiles were evaluated in a Perspex four-arm olfactometer [50]. Headspace samples (10 μL aliquots) were applied, using a micropipette (Drummond ‘microcap’, Drummond Scientific Co., Broomall, PA, USA), to a piece of filter paper (4 x 25 mm) subsequently placed in an inlet port at the end of each olfactometer arm. Gravid female parasitoids without any prior exposure to plants or hosts were transferred individually into the central chamber of the olfactometer using a custom-made piece of glass tubing. Air was drawn through the four arms towards the centre at 260 mL min^-1^. Time spent in each olfactometer arm was recorded with ‘Olfa’ software (F. Nazzi, Udine, Italy) for 12 minutes. A choice-test was carried out to compare insect responses to headspace samples from induced and control plants, as well as from oviposited and control plants. The two opposite arms held the test stimuli (10 μL aliquots of headspace sample). The remaining two arms were solvent controls. The experiment was replicated 12 times.

### Chemical analysis

Entrained VOCs were analyzed using a Hewlett-Packard 7890 GC machine (Agilent Technologies Co Ltd, Santa Clara, CA, USA) equipped with a cool-on column injector, a non-polar HP-1 capillary column (50 m, 0.32mm internal diameter, 0.52 μm film thickness) and a flame ionization detector (FID). Four μL of headspace sample was injected into the injector port of the GC instrument. Oven temperature was maintained at 30°C for 2 minutes and then programmed at 5°C min-1 to 250°C. The carrier gas was helium. Data were analyzed using HP Chemstation software. Aliquots of attractive headspace samples were analyzed using a Hewlett-Packard 5890 GC machine (Agilent Technologies) on a capillary Gas Chromatography HP-1 column (50 m, 0.32 mm internal diameter, 0.52 μm film thickness) directly coupled to a mass spectrometer (VG Autospec; Fisons Instruments, Manchester, UK) equipped with a cool on-column injector. Ionisation was performed by electron impact (70 eV at 250°C). The oven temperature was maintained at 30°C for 5 minutes, and then programmed at 5°C min^-1^ to 250°C. Tentative identifications were made by comparison of spectra with mass spectral databases [51]. Tentative identifications of the compounds were confirmed through co-injections with authentic standards. Quantification of key compounds emitted in both egg exposed (inducing), induced and control maize plants was done by comparing the peak area of these treatments to the peak area of 100 nanograms of synthetic DMNT. This was done by first injecting 1 μl of synthetic DMNT which contained 100 ng of DMNT prepared in redistilled hexane. The peak area was recorded and a response factor calculated using Eq 1 below. An equal amount of natural headspace sample containing unknown concentration of DMNT was then analysed and the amount of compound obtained using Eq 2 below.

$$\text{Response factor}=\frac{\text{Peak area}}{\text{Sample amount}}$$ Eq 1

$$\text{Amount of analyte}=\frac{\text{Peak area}}{\text{Response factor}}$$ Eq 2

### Statistical analysis

Four-arm olfactometer bioassay data, i.e. time spent in each olfactometer arm by *T*. *bournieri* and *C*. *sesamiae*, were compared by analysis of variance (ANOVA) after conversion of the data into proportions and a logratio transformation. Means were separated using Tukey’s test with α set at 0.05. Multivariate analysis of variance (MANOVA) was used to compare the quantities of volatile blends emitted by different plant treatments. Statistical analyses were done using R3.0 software [52].

## Results

### Behavioural responses of parasitoids to headspace samples of volatiles from egg exposed, neighbouring and control maize plants

**Fig 1:**
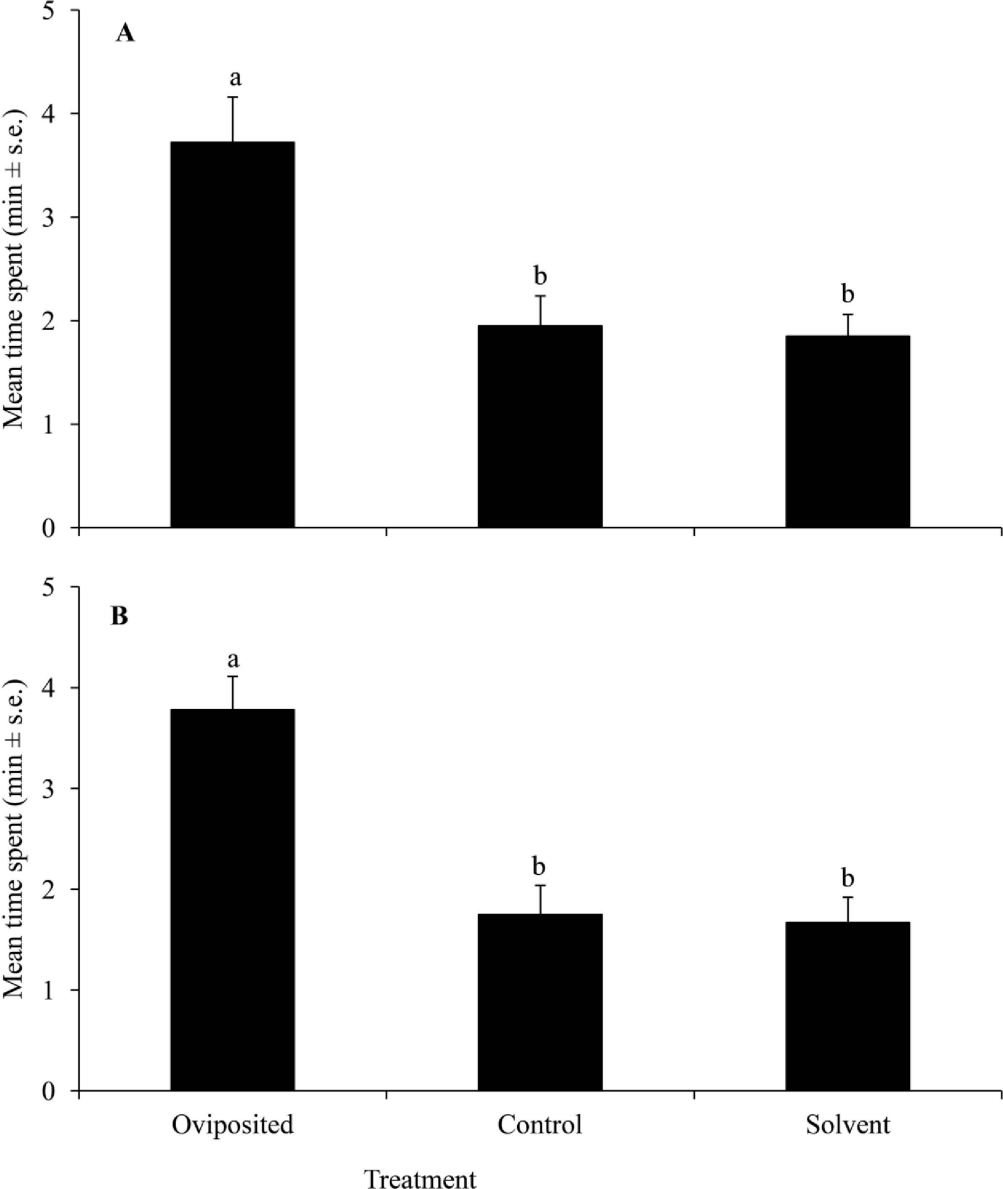
Behavioural response of female parasitoids to volatiles collected from landrace maize seedlings, ‘Nyamula’ with or without *Chilo partellus* eggs in a four-arm olfactometer bioassay. Response of (**A**) *Trichogramma bournieri*; (**B**) *Cotesia sesamiae*. Bars marked by different letters within a graph are statistically different (*P*<0.05).

**Fig 2:**
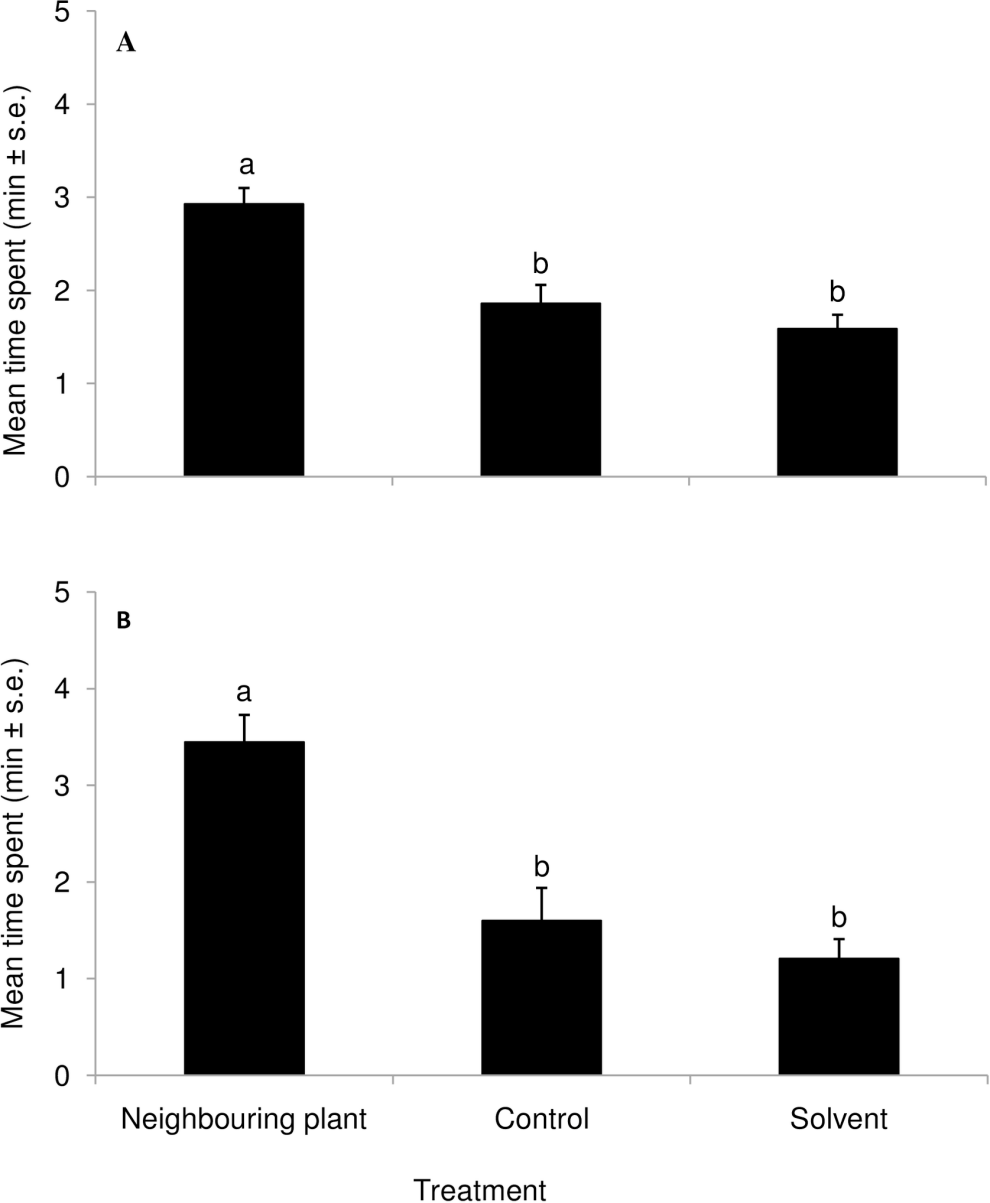
Behavioural response of parasitoids to volatiles collected from a landrace, ‘Nyamula’, neighbouring induced maize plant (exposed to maize plant emitting egg-induced volatiles) and an unexposed control plant in a four-arm olfactometer bioassay. Response of (**A**) *Trichogramma bournieri*; (**B**) *Cotesia sesamiae*. Bars marked by different letters within a graph are statistically different (*P*<0.05).

**Fig 3:**
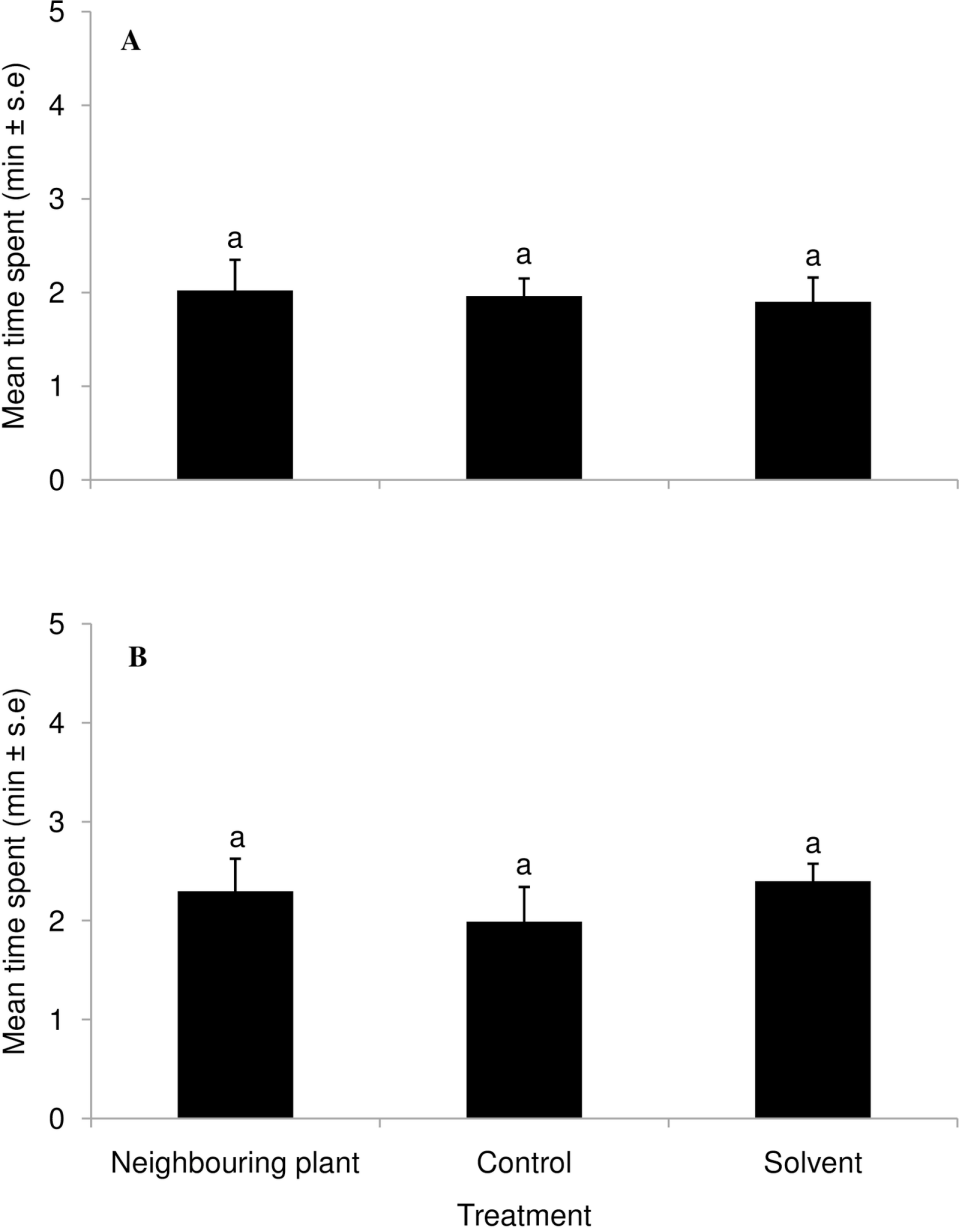
Behavioural response of parasitoids to volatiles collected from a hybrid, HB515, neighbouring induced maize plant (exposed to maize plant emitting egg-induced volatiles) and an unexposed control plant in a four-arm olfactometer bioassay. Response of (**A**) *Trichogramma bournieri*; (**B**) *Cotesia sesamiae*. Bars marked by same letters within a graph are not statistically different (*P*>0.05).

Both egg (*T*. *bournieri*) and larval (*C*. *sesamiae*) parasitoids were significantly attracted to volatiles from egg exposed landrace ‘Nyamula’ plants compared to those from plants not exposed to eggs and solvent controls (F_2,33_ = 10.37, *P*<0.001; F_2,33_ = 12.76, *P*<0.001 respectively) (Fig 1). Similarly, both egg and larval parasitoids spent significantly more time in the olfactometer arms with volatiles from neighbouring ‘Nyamula’ plants compared to arms holding volatiles from unexposed plants and solvent controls (F_2,33_ = 18.39, *P*<0.001; F_2,33_ = 10.54, *P*<0.001 respectively) (Fig 2). In contrast, there was no significant difference in time spent by both egg and larval parasitoids in arms holding volatiles from neighbouring, unexposed HB515 plants, and solvent controls (F_2,33_ = 0.12, *P*>0.05; F_2,33_ = 1.05, *P*>0.05 respectively) (Fig 3). Complete responses are shown in Tables A, B and C in S1 File attached.

### Chemical analysis

**Fig 4:**
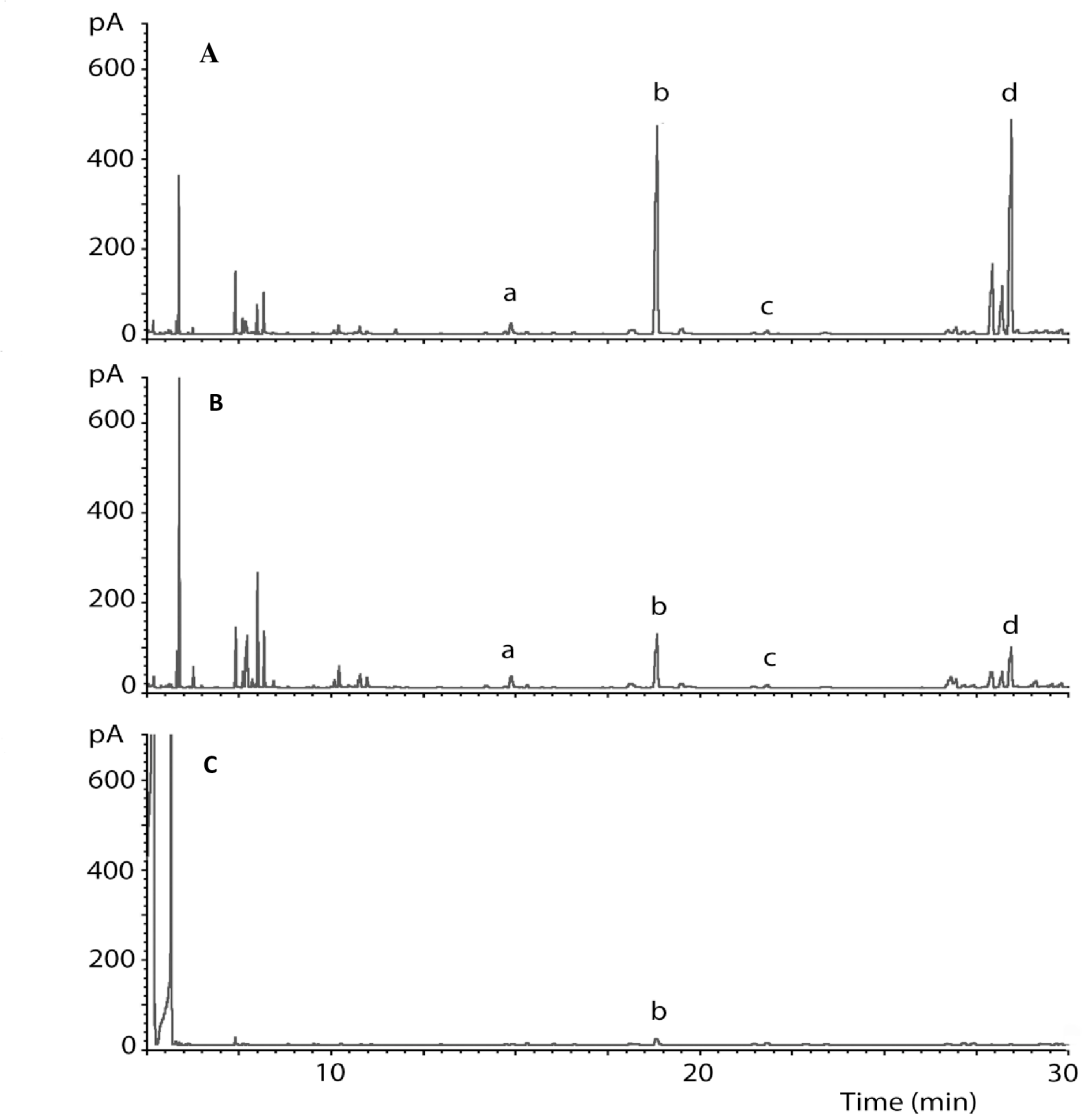
GC profiles of headspace volatiles from maize landrace, ‘Nyamula’: (**A**) exposed to oviposition, (**B**) exposed to egg-induced maize volatiles from neighbouring plant, (**C**) unexposed. The identities of some of the EAG-activec ompounds whose emission was highly elevated by oviposition and induction are as follows: (a) limonene; (b) (*E*)-4,8-dimethyl-1,3,7, nonatriene (DMNT); (c) methyl salicylate; (d) decanal.

**Fig 5:**
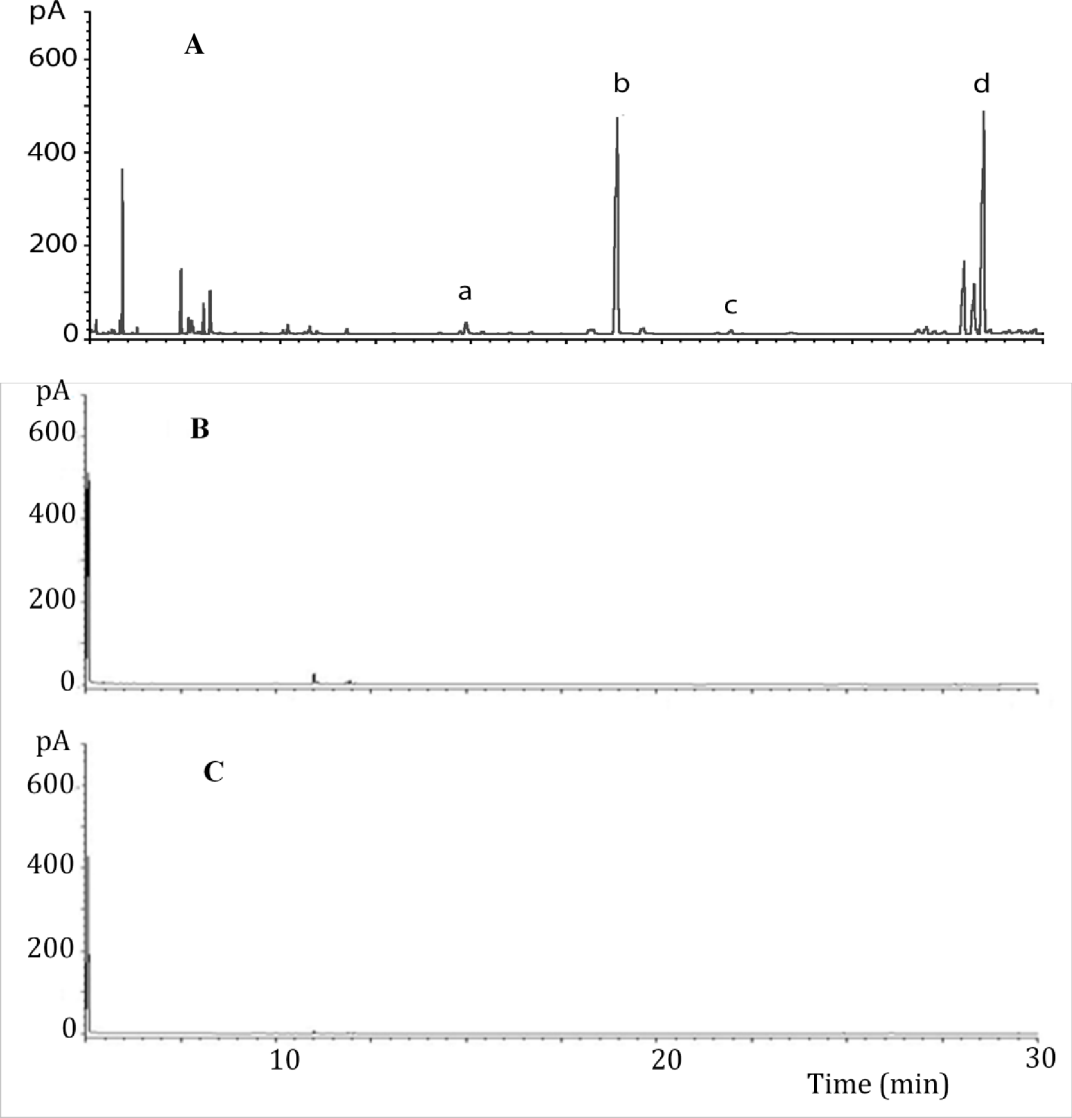
GC profiles of headspace volatiles from hybrid maize, HB515: (**A**) landrace ‘Nyamula’ exposed to oviposition used for induction, (**B**) HB515 exposed to egg-induced maize volatiles from neighbouring ‘Nyamula’ plant, (**C**) unexposed HB515 control plant. The identities of some of the EAG-activec ompounds whose emission was highly elevated by oviposition are as follows: (a) limonene; (b) (*E*)-4,8-dimethyl-1,3,7, nonatriene (DMNT); (c) methyl salicylate; (d) decanal.

Gas chromatographic analysis of the headspace samples revealed quantitative and qualitative changes in the volatile profiles emitted by plants exposed to egg deposition in comparison to unexposed control plants of the same variety. Similar observations were made for the headspace samples from the landrace ‘Nyamula’ maize plants without eggs that were exposed to volatiles from plants with eggs (Fig 4). There were marked increases in the levels of compounds which had previously been shown to be electrophysiologically active and play a key role in parasitoid attraction such as DMNT [53] in the plants with eggs and the neighbouring landrace maize plants exposed to volatiles from plants with eggs (Fig 4). However, there were no changes in the volatile profiles in hybrid maize, HB515, exposed to volatiles from maize plants with eggs (Fig 5), an indication that the difference is due to genotype. EAG active compounds in egg-induced ‘Nyamula’ are reported by [43]. Quantification of these compounds in the volatile samples in the current study showed that there were significant increases in amount of limonene, DMNT and decanal in not only the oviposited and but also in neighbouring VOC-induced landrace ‘Nyamula’ maize compared to the control (F_2,12_ = 2.10, *P*>0.05; F_2,12_ = 3.12, *P*>0.05; F_2,12_ = 0.62, *P*>0.05 respectively) (Table 1). These electrophysiologically active compounds were not detected in the hybrid maize, HB515 (Table 1).

**Table 1:** Volatile emission (ng/plant/hr) (mean ± s.e) from egg exposed (inducing), neighbouring (induced) and control maize plants (N = 5).

Compound	Inducing plant	Landrace ‘Nyamula’		Hybrid ‘HB515’	
		Induced	Control	Induced	Control
limonene	0.59 (0.28)a	0.36 (0.14)a	0.06 (0.03)b	n.d	n.d
linalool	0.53 (0.21)a	0.72 (0.20)a	0.04 (0.02)a	n.d	n.d
(*E*)-4,8-dimethyl-1,3,7-nonatriene (DMNT)	17.33 (7.44)a	8.11 (4.01)a	0.10 (0.06)b	n.d	n.d
methyl salicylate	0.42 (0.06)a	0.43 (0.07)a	0.19 (0.06)a	n.d	n.d
decanal	2.90 (2.81)a	3.35 (2.83)a	0.02 (0.02)a	n.d	n.d

Means followed by a different letter, within a row, are significantly different (multivariate analysis of variance, *P* < 0.05). *n*.*d* indicates not detected.

## Discussion

Results from the current study show that egg-induced volatiles from the maize landrace ‘Nyamula’ can induce an indirect defence response in neighbouring conspecific plants even when they are not exposed to the herbivore themselves. This suggests that egg-induced volatile organic compounds were detected and responded to by the undamaged neighbouring plants and implies that there is airborne signalling between ‘Nyamula’ plants following *C*. *partellus* egg deposition. As shown previously [43], egg deposition triggered emission of VOCs that are attractive to stemborer parasitoids but here we show the signal can be passed between plants to make the “early herbivore alert” effect precede even the physical presence of eggs on a plant. This signalling of an impending herbivore attack prompts plants to activate their defence mechanisms, eventually emitting volatile semiochemicals that attract natural enemies of the herbivores. However, the effect depended on maize genotype and was absent in the standard hybrid line HB515. Previous studies have shown that herbivore egg deposition on certain maize varieties induces emission of volatiles that are attractive to both egg and larval parasitoids. This trait has been found to be prevalent in wild and landrace maize varieties but rare in maize hybrids [19, 43, 54, 44]. The current study reports for the first time mediation of indirect defence in undamaged maize plants by egg-induced maize volatiles from neighbouring plants.

Comparing the egg-induced VOCs from the landrace maize plants and those from neighbouring intact maize plants exposed to the egg-induced VOCs revealed quantitative rather than qualitative differences. Larger quantities of VOCs were emitted from the egg exposed landrace plants compared to the VOC-induced plants. The mechanism of emission of physiologically-active volatile compounds from VOC-exposed plants can have two possible explanations. Firstly, the egg-induced maize volatiles might have been adsorbed to the surface of the VOC-exposed maize plants and later volatilized once more. Secondly, it is possible that volatiles from egg-exposed plants triggered production of electrophysiologically active semiochemicals by VOC-exposed plants. Given that VOCs have been shown to induce expression of defence genes in uninfested plants [55, 56, 23], and the fact that it is only landrace maize varieties that had been shown to respond to egg deposition and not the hybrid maize that showed changes on volatile profiles, the latter explanation seems plausible. Furthermore, hybrid maize HB515 had been shown not to respond to herbivore oviposition by activating semiochemical emissions as opposed to the landrace ‘Nyamula’ [45]. Nevertheless, additional studies are being done using labelled synthetic DMNT to determine the mechanisms of the induced responses observed in the intact neighbouring plants. The VOCs released from egg exposed and neighbouring maize plants induced by them generally matched those of previous studies on egg-induced volatiles in maize varieties [19, 43, 44]. Some of the released VOCs as well as those reported in literature include green leaf volatiles (GLVs); monoterpenes such as myrcene and limonene; homoterpenes such as (*E*)-4,8-dimethyl-1,3,7, nonatriene (DMNT); sesquiterpenes such as (*Z*)-β-farnesene and (*E*)-β-caryophyllene; phenyl propanoids including methyl salicylate and aldehydes such as decanal. DMNT, a key compound known to mediate herbivore-natural enemy interactions in these systems, was elevated by egg deposition as well as by exposure of intact maize to egg-indued VOCs. The emission of these compounds explains the observed behavioural responses of the parasitoids in the olfactometer bioassays.

Volatile organic compounds (VOCs) have been shown to induce defence responses in plants. For example, (*Z*)-3-hexenyl acetate induces defence genes in unifested leaves of Lima bean [11, 23] and *Arabidopsis* [55]. Other compounds which have been identified that elicit VOC-induced plant responses include (*Z*)-jasmone [57, 58], (*E*,*Z*)-β-ocimene, (*E*)-4,8-dimethyl-1,3,7-nonatriene (DMNT), (*E*,*E*)-4,8,12-trimethyl-1,3,7,11-tridecatetraene (TMTT) [11] and methyl salicylate [59, 60]. GLVs have also been shown to prime maize plants against subsequent herbivore attack [35]. The role of HIPVs in influencing defence pathways and responses in neighbouring undamaged plants has been previously described [27, 29, 16]. Whilst HIPVs can immediately induce defence signalling in neighbouring plants at high levels, physiologically relevant levels of HIPVs appear to prime plants to prepare for future herbivore and pathogen attack [31]. Since most of these volatile compounds had elevated levels and emissions following egg deposition in the current study, it is suggested that they are the ones responsible for the induction of indirect defence mechanisms in neighbouring undamaged maize plants.

Plants that are able to ‘eavesdrop’ from neighbouring attacked plants and use these cues to activate and tailor their defences according to information derived from their attacked neighbours in anticipation of herbivore attack may gain a selective advantage over plants that are unable to make use of these signal cues [23]. Additionally, egg-induced volatiles provide natural enemies of herbivores with early-alert cues indicating the presence of hosts [42, 61]. Thus, the emission of volatile organic compounds attractive to natural enemies of herbivores from both the egg exposed and neighbouring undamaged maize plants increases the signal strength of these attractive cues thereby increasing the recruitment and foraging efficiency of the antagonists. This eventually increases the preventive defence strategy of the plant community against herbivores since large numbers of natural enemies are recruited to parasitize eggs as well as emerging larvae before causing damage to the plants [41]. Furthermore, parasitized lepidopteran eggs do not develop into larvae and parasitised larvae feed less than non-parasitized ones and die upon emergence of the adult wasp, which greatly reduces damage to the plant [62, 63].

The induction of defensive responses in maize plants through airborne signals from egg exposed plants even before any damage is inflicted on the crop plant could contribute to natural protection of crop plants against stemborers. For instance, intercropping maize varieties that are able to respond defensively to early-herbivory with varieties that can perceive the emitted chemical signals can enhance natural enemy recruitment as opposed to crop monocultures that cannot respond to egg deposition or volatile signals induced by oviposition. This could be of practical importance as demonstrated by Pettersson *et al*. [64] and Ninkovic *et al*. [65] who planted mixtures of barley cultivars emitting volatiles that negatively affected host plant acceptance by aphids. Since there is variability in emission of egg-induced semiochemicals in maize germplasm, and the levels of emission may be too low for practical crop protection purposes, it may be possible to boost the strength of the signal by companion planting with maize varieties that emit larger amounts of effective volatile compounds as described by Pickett *et al*. [66].

## Supporting Information

S1 File Tables A, B and C.
(XLS)

## References

[1] Kessler A, Baldwin IT. Plant responses to insect herbivory: the emerging molecular analysis. Annu. Rev. Plant Biol. 2002; 53:299–28. 12221978 10.1146/annurev.arplant.53.100301.135207

[2] Pieterse CMJ, Dicke M. Plant interactions with microbes and insects: from molecular mechanisms to ecology. Trends Plant Sci. 2007; 12:564–69. 17997347 10.1016/j.tplants.2007.09.004

[3] Schaller A. (eds.) Induced Plant Resistance to Herbivory, Springer, Berlin, Germany 2008.

[4] Dicke M, van Loon JJA, Soler R. Chemical complexity of volatiles from plants induced by multiple attack. Nat. Chem. Biol. 2009; 5:317–24. 10.1038/nchembio.169 19377458

[5] Khan ZR, Midega CAO, Bruce TJA, Hooper AM, Pickett JA. Exploiting phytochemicals for developing a push-pull crop protection strategy for cereal farmers in Africa. J. Exp. Bot. 2010; 61:4185–96. 10.1093/jxb/erq229 20670998

[6] Roda AL, Baldwin IT. Molecular technology reveals how the induced direct defenses of plants work. Basic Appl. Ecol. 2003; 4: 15–26.

[7] Mutyambai DM, Midega CAO, Bruce TJA, Van den Berg J, Pickett J, Khan ZR. Behaviour and biology of *Chilo partellus* on maize landraces. Entomol. Exp. Appl. 2014; 153:170–181.

[8] Karban R, Baldwin IT, Baxter KJ, Laue G, Felton GW. Communication between plants: induced resistance in wild tobacco plants following clipping of neighbouring sagebrush. Oecol. 2000; 125:66–71. 10.1007/PL00008892 28308223

[9] Kessler A, Baldwin IT. Defensive function of herbivore-induced plant volatile emissions in nature. Science 2001; 291:2141–44. 11251117 10.1126/science.291.5511.2141

[10] Baldwin IT, Schultz JC. Rapid changes in tree leaf chemistry induced by damage: evidence of communication between plants. Science 1983; 221:277–79. 17815197 10.1126/science.221.4607.277

[11] Arimura G, Ozawa R, Shimoda T, Nishioka T, Boland W, Takabayashi J. Herbivory-induced volatiles elicit defence genes in Lima bean. Nature 2000; 406:512–15. 10952311 10.1038/35020072

[12] Karban R, Maron J. The fitness consequences of interspecific eavesdropping between plants. Ecology 2002; 83:1209–1213.

[13] Heil M. Indirect defence through tritrophic interactions. New Phytol. 2008; 178:41–61. 18086230 10.1111/j.1469-8137.2007.02330.x

[14] Arimura G, Matsui K Takabayashi J. Chemical and molecular ecology of herbivore-induced plant volatiles: proximate factors and their ultimate functions. Plant Cell Physiol. 2009; 50:911–23. 10.1093/pcp/pcp030 19246460

[15] Karban R. The ecology and evolution of induced resistance against herbivores. Funct. Ecol. 2011; 25:339–47.

[16] Chamberlain K. Airborne plant-plant communication. Skvortsovia 2014; 1:112–32.

[17] Rodriguez-Saona CR, Rodriguez-Saona LE, Frost CJ. Herbivore-induced volatiles in the perennial shrub, *Vaccinium corymbosum* and their role in interbranch signalling. J. Chem. Ecol. 2009; 35:163–75. 10.1007/s10886-008-9579-z 19159981

[18] War AR, Sharma HC, Paulraj MG, War MY, Ignacimuthu S. Herbivore-induced plant volatiles: Their role in plant defence for pest management. Plant Signal Behav. 2011; 6:1973–1978. 22105032 10.4161/psb.6.12.18053 PMC3337190

[19] Tamiru A, Bruce TJA, Woodcock CM, Caulifield CJ, Midega CAO, Ogol CKPO. Maize landraces recruit egg and larval parasitoids in response to egg deposition by a herbivore. Ecol. Lett. 2011; 14:1075–1083. 10.1111/j.1461-0248.2011.01674.x 21831133

[20] Takabayashi J, Dicke M, Posthumus M. Variation in composition of predator-attracting allelochemicals emitted by herbivore-infested plants: relative influence of plant and herbivore. Chemoecol. 1991; 2:1–6.

[21] Turlings TCJ, Bernasconi M, Bertossa R, Bigler F, Caloz G, Dorn S. The induction of volatile emissions in maize by three herbivore species with different feeding habits-possible consequences of their natural enemies. Biol Control 1998; 11:122–129.

[22] Gouinguené S, Degen T, Turlings TCJ. Variability in herbivore-induced odour emissions among maize cultivars and their wild ancestors (teosinte). Chemoecology 2001; 11:9–16.

[23] Kost C, Heil M. Herbivore-induced plant volatiles induce an indirect defence in neighbouring plants. J. Ecol. 2006; 94:619–28.

[24] Fowler SV, Lawton JH. Rapidly induced defences and talking trees: the devil’s advocate position. Amer. Nat. 1985; 126:181–95.

[25] Bruin J, Dicke M, Sabelis MW. Plants are better protected against spider-mites after exposure to volatiles from infested conspecifics. Experientia 1992; 48:525–29.

[26] Shonle I, Bergelson J. Interplant communication revisited. Ecology 1995; 76:2660–63.

[27] Chamberlain K, Pickett JA, Woodcock CM. Plant signalling and induced defence in insect attack. Mol. Plant. Pathol. 2000; 1:67–72. 10.1046/j.1364-3703.2000.00009.x 20572952

[28] Chamberlain K, Guerrieri E, Pennacchio F, Petersson J, Pickett JA, Poppy GM, et al Can aphid-induced plant signals be transmitted aerially and through the rhizosphere. Biochem. Sys. Ecol. 2001; 29:1063–74.

[29] Pickett JA, Poppy GM. Switching on plant genes by external chemical signals. Trends Plant Sci. 2001; 6:137–39. 11286900 10.1016/s1360-1385(01)01899-4

[30] Tscharntke T, Thiessen S, Dolch R, Boland W. Herbivory, induced resistance and interplant signal transfer in *Alnus glutinosa*. Biochem. Sys. Ecol. 2001; 29:1025–1047.

[31] Heil M, Ton J. Long distance signalling in plant defence. Trends Plant Sci. 2008; 13:264–72. 10.1016/j.tplants.2008.03.005 18487073

[32] Gomi K, Yamasaki Y, Yamamoto H, Akimisu K. Characterization of a hydroperoxide lyase gene and effect of C_6_-volaties on expression of gene of the oxylipin metabolism in *Citrus*. J. Plant Physiol. 2003; 160:1129–31. 10.1078/0176-1617-01177 14610891

[33] Paschold A, Halitschke R, Baldwin IT. Using ‘mute’ plants to translate volatile signals. Plant J. 2006; 45:275–291. 16367970 10.1111/j.1365-313X.2005.02623.x

[34] Arimura G, Ozawa R, Nishioka T, Boland W, Koch T, Kuhnemann F, et al Herbivore-induced volatiles induce the emission of ethylene in neighbouring Lima bean plants. Plant J. 2002; 29:87–98. 12060229 10.1046/j.1365-313x.2002.01198.x

[35] Engelberth J, Alborn HT, Schmelz EA, Tumlinson JH. Airborne signals prime plants against insect herbivore attack. Proc. Natl. Acad. Sci. USA 2004; 101:1781–85. 14749516 10.1073/pnas.0308037100 PMC341853

[36] Ruther J, Kleir S. Plant-plant signalling: ethylene synergizes volatile emission in *Zea mays* induced by exposure to (*Z*)-3-hexen-1-ol. J. Chem. Ecol. 2005; 31:2217–22. 16132223 10.1007/s10886-005-6413-8

[37] Kfir R, Overholt WW, Khan ZR, Polaszek A. Biology and management of economically important lepidopteran cereal stem borers in Africa. Ann. Rev. Entom. 2002; 47:701–731. 11729089 10.1146/annurev.ento.47.091201.145254

[38] Van den Berg J, Nur AF. Chemical control In: African Cereal Stemborers: Economic Importance, Taxonomy, Natural Enemies and Control (ed. Polaszek A.). CABI, Wellingford, 1998; pp. 530.

[39] Van den Berg J, Nur AF, Polaszek A. Cultural control In Polaszek A. African Cereal Stem Borers: Economic Importance, Taxonomy, Natural Enemies and Control. Wallingford, UK: CABI 1998; pp. 333–347.

[40] Karban R, Baldwin IT. Induced Responses to Herbivory. Chicago: Chicago University Press; 1997 p 319.

[41] Hilker M, Meiners T. Induction of plant responses to oviposition and feeding by herbivorous arthropods: a comparison. Entomol. Exp. Appl. 2002;104:181–92.

[42] Hilker M, Meiners T. Early herbivore alert: insect eggs induce plant defense. J. Chem. Ecol. 2006; 32:1379–97. 16718566 10.1007/s10886-006-9057-4

[43] Tamiru A, Bruce TJA, Midega CAO, Woodcock CM, Birkett MA, Pickett JA, et al Oviposition induced volatile emissions from African smallholder farmers’ maize varieties. J. Chem. Ecol. 2012; 38:232–34. 10.1007/s10886-012-0082-1 22367424

[44] Mutyambai DM, Bruce TJA, Midega CAO, Woodcock CM, Caulfield JC, Van den Berg J, et al Response of parasitoids to volatiles induced by *Chilo partellus* oviposition on teosinte, a wild ancestor of maize. J. Chem. Ecol. 2015a; 41:323–29. 10.1007/s10886-015-0570-1 25943860 PMC4427631

[45] Mutyambai DM. Exploiting early herbivory-induced defense traits in *Zea* species for the management of *Chilo partellus* in East Africa [PhD thesis]. North West University, Potchefstroom, South Africa; 2015b.

[46] Penaflor MFGV, Erb M, Robert CAM, Miranda LA, Werneburg AG, Dossi FCA, et al Oviposition by a moth suppresses constitutive and herbivore induced plant volatiles in maize. Planta 2011; 234:207–215. 10.1007/s00425-011-1409-9 21509694

[47] Ochieng RS, Onyango FO, Bungu MDO. Improvement of techniques for mass-culture of *Chilo partellus* (Swinhoe). Insect Sci. Appl. 1985; 6:425–28.

[48] Overholt WA, Ochieng JO, Lammers P, Ogedah K. Rearing and field release methods for *Cotesia flavipes* Cameron (Hymenoptera: Braconidae), a parasitoid of tropical gramineous stem borers. Insect Sci. Appl. 1994; 15:253–59.

[49] Agelopoulos NG, Hooper AM, Maniar SP, Pickett JA Wadhams LJ. A novel approach for isolation of volatile chemicals released by individual leaves of a plant in situ. J Chem Ecol. 1999; 25:1411–25.

[50] Pettersson J. An aphid sex attractant 1. Biological studies. Entomol. Scand. 1970; 1:63–73.

[51] Nist. NIST mass spectral search for the NIST/EPA/NIH mass spectral library version 2.0. office of the Standard Reference Data Base, National Institute of Standards and Technology, Gaithersburg, Maryland; 2005.

[52] R3.0.The R Foundation for Statistical Computing Platform, Vienna, Austria; 2013.

[53] Khan ZR, Ampong-Nyarko K, Chilishwa P, Hassanali A, Kimani S, Lwande W, et al Intercropping increases parasitism of pests. Nature 1997; 388:631–32.

[54] Tamiru A, Khan ZR, Bruce TJA. New directions for improving crop resistance to insects by breeding for egg induced defence. Curr. Opin. Insect Sci. 2015; 9:1–5. 10.1016/j.cois.2015.02.011 32846708

[55] Bate NJ, Rothstein SJ. C6-volatile derived from the lopoxygenase pathway induce a subset of defence-related genes. Plant J. 1998; 16:561–69. 10036774 10.1046/j.1365-313x.1998.00324.x

[56] Arimura G, Ozawa R, Horiuchi J, Nishioka T, Takabayashi J. Plant-plant interactions mediated by volatiles emitted from plants infested by spider mites. Biochem. Syst. Ecol. 2001; 29:1049–61.

[57] Birkett MA, Campbell CAM, Chamberlain K, Guerrieri E, Hick AJ, Martin JL, et al New roles of *cis*-jasmone as an insect semiochemical and in plant defence. Proc. Natl. Acad. Sci. USA 2000; 97:9329–34. 10900270 10.1073/pnas.160241697 PMC16867

[58] Bruce TJA, Martin JL, Pickett JA, Pye BJ, Smart LE, Wadhams LJ. *Cis*-jasmone treatment induces resistance in wheat plants against the grain aphid, *Sitobion avenae* (Fabricius) (Homoptera: Aphidae). Pest Manag Sci. 2003; 59:1031–36. 12974355 10.1002/ps.730

[59] Shulaev V, Silverman P, Raskin I. Airborne signalling by methyl salicylate in plant pathogen resistance. Nature 1997; 385:718–21.

[60] Giron-Calva P, Molina-Torres J, Heil M. Volatile Dose and Exposure Time Impact Perception in Neighboring Plants. J. Chem. Ecol. 2012; 38: 226–28. 10.1007/s10886-012-0072-3 22327276

[61] Bruce TJA, Midega CAO, Birkett MA, Pickett JA, Khan ZR. Is quality more important than quantity? Insect behavioural responses to changes in a volatile blend after stemborer oviposition on an African grass. Biol. Lett. 2010; 6:314–17. 10.1098/rsbl.2009.0953 20031982 PMC2880062

[62] Hoballah FME, Tamo C, Turlings TCJ. Differential attractiveness of induced odours emitted by eight maize varieties for the parasitoid *Cotesia marginiventris*: Is quality or quantity important? J. Chem. Ecol. 2002; 28:951–68. 12049233 10.1023/a:1015253600083

[63] Hoballah FME, Köllner TG, Degenhardt J, Turlings TCJ. Costs of induced volatile production in maize. Oikos 2004; 105:168–80.

[64] Pettersson J, Ninkovic V, Ahmed A, Volatiles from different barley cultivars affect aphid acceptance of neighbouring plants. Acta Agric. Scand. Sect. B Soil Plant Sci. 1999; 49:152–157.

[65] Ninkovic V, Olsson U, Pettersson J. Mixing barley cultivars affects aphid host plant acceptance in field experiments. Entomol. Exp. Appl. 2002; 102:177–82.

[66] Pickett JA, Woodcock CM, Midega CAO, Khan ZR. Push-pull farming systems. Curr. Opin. Biotechnol. 2014; 26:125–132. 10.1016/j.copbio.2013.12.006 24445079

